# The Influence of the Chemical Composition of Beeswax Foundation Sheets on Their Acceptability by the Bee’s Colony

**DOI:** 10.3390/molecules29235489

**Published:** 2024-11-21

**Authors:** Sava Ledjanac, Fatjon Hoxha, Nebojša Jasnić, Aleksandra Tasić, Marko Jovanović, Slavica Blagojević, Nada Plavša, Tomislav Tosti

**Affiliations:** 1 Faculty of Agriculture, University of Novi Sad, Trg Dositeja Obradovića 8, 21000 Novi Sad, Serbia; sava_apis@yahoo.com (S.L.); plavsa.nada@gmail.com (N.P.); 2 Faculty of Biotechnology and Food, Agricultural University of Tirana, ‘Paisi Vodica’ Street, Koder-Kamez, 1029 Tirana, Albania; fhoxha@ubt.edu.al; 3 Faculty of Biology, University of Belgrade, Studentski Trg 16, 11000 Belgrade, Serbia; jasnicn@bio.bg.ac.rs; 4 Scientific Institute of Veterinary Medicine of Serbia, Janisa Janulisa 14, 11000 Belgrade, Serbia; alekstasic79@gmail.com; 5 Institute of General and Physical Chemistry, Studentski Trg 12/V, P.O. Box 45, 11158 Belgrade, Serbia; markspace711@gmail.com; 6 Faculty of Pharmacy, University of Belgrade, Vojvode Stepe 450, 11221 Belgrade, Serbia; slavica.blagojevic@pharmacy.bg.ac.rs; 7 Institute of Chemistry, Technology and Metallurgy, National Institute of the Republic of Serbia, University of Belgrade, Studentski Trg 12-16, 11158 Belgrade, Serbia

**Keywords:** bee’s colony, acceptability, honeycomb, beeswax, paraffin

## Abstract

Beeswax is one of the most important products for the well-being of bee colonies. The wax glands of young worker bees produce beeswax, which serves as a building material for honeycomb construction. Beekeepers using hives with mobile frames mainly utilize local beeswax to make foundations. Any paraffin addition represents adulteration, resulting in a high degree of contamination. During the preparation of re-used beeswax, losses during the process may instigate producers to add cheaper, wax-like substances like paraffin and tallow. This article presents a systematic investigation of the quality of beeswax foundation from six major producers in Vojvodina, Serbia, by applying the classic analytical procedure for the determination of selected physicochemical parameters and instrumental gas chromatography coupled with mass spectrometry (GC–MS) and Fourier transform infrared attenuated total reflection (FTIR–ATR) spectroscopy techniques. FTIR–ATR detected possible paraffin and beef tallow in 72 foundation sheet samples. This technique was complemented with GC–MS. This analysis revealed that paraffin content ranged between 19.75 and 85.68%, while no tallow was detected over the two-year period. Two sheets from each manufacturer were placed into wired Langstroth–Ruth frames and placed in beehives. The construction, based on built cells, was monitored every 24 h. Evaluating newly inserted sheets proved that without quality nectar, there is no intensive building, regardless of adulteration.

## 1. Introduction

Beeswax, a complex chemical product of the glands of young 12- to 18-days-old worker bees, *Apis mellifera* L., *Fam. Apidae*, serves as a construction material for comb building [1,2]. The construction of combs and the potential ability of bees to produce wax are closely related to the physiological state of the glands that secrete wax, the state of the colony, and external environment factors (mainly the presence of nectar and pollen in nature). Glands for secreting wax, consisting of glandular cells of hypodermal origin, are located on the inner side of the fourth to seventh abdominal sternites of *Apis mellifera* worker bees [3]. These glands are absent in the queen and the drones. In the life of bees, wax has a very important role, because it affects the production of honey and the reproduction of bees. The queen lays her eggs in specific hexagon-shaped cells that are about 0.25 mm thick and 5 to 6 mm in diameter for worker bees, whereas future queens and drones have slightly wider cells. Besides offspring laying, a portion of the comb is used as storage for pollen and bee bread, which they consume in addition to honey [2].

Chemically, beeswax is a lipid-based complex mixture of saturated and unsaturated linear and complex monoesters, hydrocarbons, free fatty acids, free fatty alcohols, and other minor substances produced by the worker honeybee [4,5]. Pure beeswax is the product of *Apis mellifera*, containing over 300 different substances [6]. Additionally, beeswax has a pleasant honey-like aroma, which is attributed to approximately 50 different aroma compounds contributing to its distinctive scent [2,7]. The concentration of individual components and substance classes in natural beeswax may vary depending on the species and genetics of the bees, the age of the wax, geographical origin, and climatic conditions of its production [4]. It has been reported that there are no significant differences in the basic chemical composition of authentic wax originating from different *Apis mellifera* subspecies [2,5]. In general terms, the total content of predominate chemical classes of components in the beeswax of *Apis mellifera* varies (in % (*w*/*w*)); these chemical classes include linear wax monoesters and hydroxymonoesters with chain lengths of C_40_–C_48_ (35–45), complex wax esters containing 15–hydroxypalmitic acid or diols (15–27), aliphatic hydrocarbons with a predominant chain length of C_27_–C_33_ (12.0–17.8), and free fatty acids, including the most saturated fatty acids with a chain length of C_24_–C_32_ (12–14) [2,3,4,5,6,8,9,10,11,12].

Beeswax is insoluble in water and resistant to many acids, making it an ideal material for protective coatings and barrier applications. However, it is soluble in most organic solvents, such as ether, benzene, chloroform, turpentine oil, and, after heating, in alcohol and fatty acids [13].

The wax is hard, with a granular fracture, opaque, and has a pleasant smell reminiscent of honey. It becomes plastic when kneaded and does not smear under the fingers. The taste of the wax is weak, although it is characteristic in its own way. Beekeepers who use frame hives use most of their local beeswax production to make wax bases, as printed sheets of wax that are given to the bees as guides for building new combs. Specialized manufacturers usually make printed panels of foundation sheets, since a special roller press is required to print the honeycomb pattern [13]. The honeycomb that bees use should be of a high quality, with properly built cells, and without a large number of drones and deformed cells. The strength of the comb depends on the quality of the foundation sheets, their thickness, the wax quality, and the storage length. A comb built on foundation sheets is stronger than a natural one, because the sheet of the honeycomb foundation is thicker, and the partitions are stronger than those in natural honeycomb [13].

High-quality companies stock fertilized queens, bees, and brood of all ages that are used for making honeycombs. Colonies must be supplied with food such as carbohydrates and proteins. When the conditions are adequate for the construction of combs, bees begin to nurture 3 to 4 times more brood, and the ability of bees to secrete wax and make combs increases proportionally to the number of nurtured broods, since these are mutually related actions [14,15]. The more frames with foundations built by the bees, the more it will be possible to remove old, unsuitable combs, which is the natural cycle of beeswax [14,15].

In the complete absence of natural food sources, bee colonies must be fed in a simulative way with carbohydrate food (50% sugar solution). Nutrition ensures the secretion of wax and the construction of combs and enables an increase in the number of bees by an average of 15%. Feeding is done daily or every other day with 0.3–0.5 kg of sugar. In periods of favorable weather conditions, the foundation sheets are built by the bees in the fastest way and with the highest quality, possibly when they are placed between the frames with the brood. As the comb is built, new frames with foundations are gradually added to the colonies. Under good secreting conditions, strong colonies build more combs than they need to expand the nest [14,15].

Beeswax is a natural product, and the presence of any additive is not allowed. Due to the high demand for and price of beeswax, unscrupulous manufacturers often mix it with other cheaper and wax-like substances (adulterants), such as paraffin, ceresin, tallow, and carnauba wax, amongst others, increasing the profit. Such examples of counterfeiting are very common, not only due to the insufficient domestic production of beeswax but also the lack of mandatory legal regulations concerning the quality of beeswax. The production of artificial wax is prohibited by law; any addition of hydrocarbons from paraffins and microcrystalline waxes, triglycerides from palmitic acid, fat and hardened beef tallow, industrially produced fatty acids (palmitic, stearic acid), long-chain alcohols (C_16_–C_18_), or synthetic esters (C_32_–C_36_) is considered as adulteration and contamination [1,2].

The quality criteria for beeswax are defined by physicochemical parameters such as the melting point (MP), acid number (AN), ester number (EN), ester/acid ratio (EN/AN), saponification number (SN), relative density, water content, refractive index at 75 °C, peroxide value, and iodine number [1]. However, the melting point; acid, saponification, and ester numbers (value); and the ester/acid ratio are physicochemical parameters commonly used to evaluate beeswax purity and distinguish possible adulterations [2,3,6,11,16,17,18,19].

The chemical composition of beeswax is determined by its botanical and geographical origins, and the MP of beeswax, which is determined by the capillary tube method [16] and ring method (drop point (DP) [20], is not constant and varies slightly. The drop point (Section 4.5.1) represents the temperature at which ball of a molten beeswax passes through a ring. Various pharmacopoeias state that the MP value of beeswax ranges from 61 to 65 °C [2] and 61 to 66 °C [20] but is most commonly 62–65 °C [11]. When beeswax is adulterated with paraffins of a low MP, the melting point of beeswax decreases, indicating the presence of adulterants [11,16].

The acid and ester number (value) measure the free fatty acids and esterified fatty acids present in beeswax, respectively (Section 4.5.2 and Section 4.5.4), and provide insight into the chemical composition of the beeswax. Although the presence of other acidic substances can affect the acid number, calculating the acid number is a simple method for the determination of the presence of beeswax adulteration, because the acid number decreases with the addition of paraffin and increases with the addition of stearic acid [3]. The ester/acid ratio gives information about whether natural beeswax has changed significantly due to prolonged or excessive heating. The saponification number (value) reflects the amount of total fatty acids, either free or esterified, present in beeswax (Section 4.5.3). In relation to established values, the saponification number decreases when the beeswax is adulterated with paraffins and increases when beef tallow or stearic acid is used as an adulterant. Based on the *European Pharmacopoeia* [20], the ranges of AN, EN, and SN values, expressed in mg KOH g^−1^, are 17–22, 70–80, and 87–102, respectively, while the range of the ester/acid ratio, based on the International Honey Commission (IHC), is 3.3–4.3 [2].

The addition of foreign substances such as paraffin, beef tallow, and stearic acid, which are commonly used for the adulteration of beeswax, degrades the physicochemical characteristics of the beeswax, reducing the quality of beeswax as well as the quality of bases that are made from such adulterated beeswax [1,21]. When small amounts of adulterants are added to beeswax, the classical quality physicochemical analytical methods do not always detect the quantities of these adulterants (e.g., paraffin) [17], and hence prove to be inadequate for a reliable measure of beeswax purity [1,11,12,16]. In fact, these can only provide an indication of possible adulteration. For reliable detection of the most common adulterants in beeswax, physicochemical methods should be verified and complemented with more reliable and sensitive gas chromatography (GC) and spectroscopy techniques. GC with different detectors, Fourier transform infrared (FTIR), and Nuclear Magnetic Resonance (NMR) spectroscopy are used to distinguish between authentic beeswax and beeswax adulterated by small quantities (about or less than 5%) of paraffin, ceresin, stearin, and other natural waxes and fats (e.g., beef tallow).

For the chemical characterization and determination of individual components of beeswax, the quality control, identification, and quantification of adulterants in beeswax are carried out using GC combined with mass spectrometry (GC–MS) [5,8,21,22,23,24,25,26] and high-temperature gas chromatography coupled with a flame ionization detector (HTGC–FID) [9,10,11,12,27]. The added adulterant can be identified based on the differences between the number of selected components, such as hydrocarbons, fatty acids, and monoesters, in pure and adulterated beeswax and the presence of non-natural beeswax components caused by the adulteration of the beeswax. Unlike adulterants (e.g., paraffin and ceresin), which contain hydrocarbons in a linear sequence with even and odd numbers of carbon atoms, natural waxes contain n-alkanes with an odd number of carbon atoms, while hydrocarbons with an even number are present in traces. In GC detection, the adulteration of beeswax with paraffin can be simply ascertained from chromatograms as an increase in chromatographic peak heights of even-numbered n-alkanes [9,10,11,21,23,26,27] and the presence of n-alkanes containing over 35 carbon atoms, which are not characteristic of pure beeswax [23]. The presence of stearic, myristic, palmitic, and oleic acids is confirmation that the wax is adulterated with beef tallow [27]. The addition of free stearic acid to wax was successfully determined at very low concentrations (<1%), because it was not detected in pure beeswax samples [27]. The minimum estimated percent of paraffin, detected using GC–MS, was 3% [23]. HTGC–FID can detect the presence of paraffin, carnauba, stearic acid, and tallow in pure beeswax from *Apis mellifera* in very low concentrations (1–4%) [27].

Fourier transform infrared (FTIR) spectroscopy combined with attenuated total reflection (ATR) (the FTIR–ATR technique) has been recently described as an alternative approach to GC that uses detectors which have been widely used in the last two decades for the chemical characterization of beeswax [17,18,19,24,28,29,30,31]. FTIR–ATR analysis, which includes a procedure for the quantification of adulterants in contaminated beeswax samples, is focused on the absorbance peak shift in the infrared spectra that is possibly a response to the changes in the natural composition of beeswax in the presence of adulterants. The FTIR–ATR method for the detection of multicomponent adulteration of beeswax was elaborated by Tanner and Lichtenberg–Kraag, who proved that in situations where adulteration is carried out with more than five substances (paraffin, stearic acid, tallow, carnauba wax, and candelilla wax), they could determine this with the same accuracy as when one adulterant is present [29]. Using FTIR–ATR spectroscopy, it is possible to detect the adulteration of beeswax with a detection limit of approximately 5% [28,31] or 3% [17] of paraffin, beef tallow, stearic acid, and carnauba wax as adulterants and their combinations [29].

Comb-shaped beeswax is a natural material for packing honey. Despite coming into contact with honey used as food, beeswax used in beekeeping to produce honeycombs is not subject to mandatory quality control measures before being released on the market [3]. Quality control measures are applied for beeswax used as a food additive E901 [4] or as a pharmaceutical product (the Council of Europe, 2020). European legislation defines beeswax and implicitly honeycomb as “bee products” that are used in beekeeping (Regulation (EC) No 1774/2002) [32] and categorizes beeswax as a product not intended for human consumption (Commission Regulation (EU) No 142/2011) [33]. This prohibits the importation and transit through the EU of beeswax in the form of honeycomb (Chapter VIII, Art 25 of Commission Regulation (EU) No 142/2011).

The aim of this study was to investigate the occurrence of counterfeit substances, such as paraffin and beef tallow, in wax foundations available on the Vojvodina market, which are shipped worldwide, and to assess how these substances impact the acceptance of wax foundations by bees.

## 2. Results

### 2.1. Results of Wax Quality Assessment Based on Physicochemical Parameters

The determination of the physicochemical parameters (drop point, acid, saponification, and ester number, ester/acid ratio, content of paraffin and beef tallow) of the beeswax samples was performed as the starting point of our investigation. Based on the well-known fact that paraffin and beef tallow are the most common adulterants, their physicochemical properties were analyzed. The results are presented in Table 1.

The results depicted in Table 1 clearly show that most sensitive factors for determining paraffin adulteration were the acid, ester, and saponification numbers. Also, we can conclude that as paraffin content increases, the acid, ester, and saponification numbers decrease. This is expected, because paraffin does not contain free or esterified fatty acids. On the other hand, the ester/acid ratio was not affected by the paraffin adulteration, and its value remained the same as in pure beeswax. An explanation for this phenomenon could be that the values of the ester and acid numbers were equally reduced; hence, their ratio remained the same. The values of the drop point showed that there was not a correlation between paraffin content and the drop point.

FTIR–ATR characterization was used for the analysis of paraffin content in beeswax samples. FTIR–ATR (disturbed total reflection with Fourier transform) is a sensitive, fast, simple, and non-destructive method that allows testing of the quality of waxes without prior sample preparation. The values of paraffin content obtained in the tested samples are shown in Table 2.

The fingerprint region in the spectrum of beeswax is located in the range ṽ: 1800–1000 cm^−1^. The characteristic bands that appeared in it originate from the vibrations of the C=O bond.

Between 3000 and 2800 cm^−1^, bands originating from symmetric and asymmetric C–H stretching vibrations were detected. These bands were due to the presence of hydrocarbons. The adulteration of beeswax with paraffin results in reduced absorption of IR light in the “fingerprint” region [17]. The addition of paraffin to the beeswax manifests itself through a change in the intensity ratio of the absorption bands (1739/2852 cm^−1^ and 1714/2852 cm^−1^), which was successfully used to determine its content. When the wax was adulterated with fats of animal origin, the band originating from the valence vibrations of the C=O bond of the carboxyl group of the triglyceride ester (1745 cm^−1^) appeared, and it was significantly more intense than the one characteristic of pure beeswax. The addition of stearic acid to beeswax manifested itself in the increasing band at the wavelength of 1711 cm^−1^, which originates from the C=O valence vibrations of the carboxyl group of fatty acids [17,18].

### 2.2. Gas Chromatography Analysis of the Beeswax Samples

**Figure 1:**
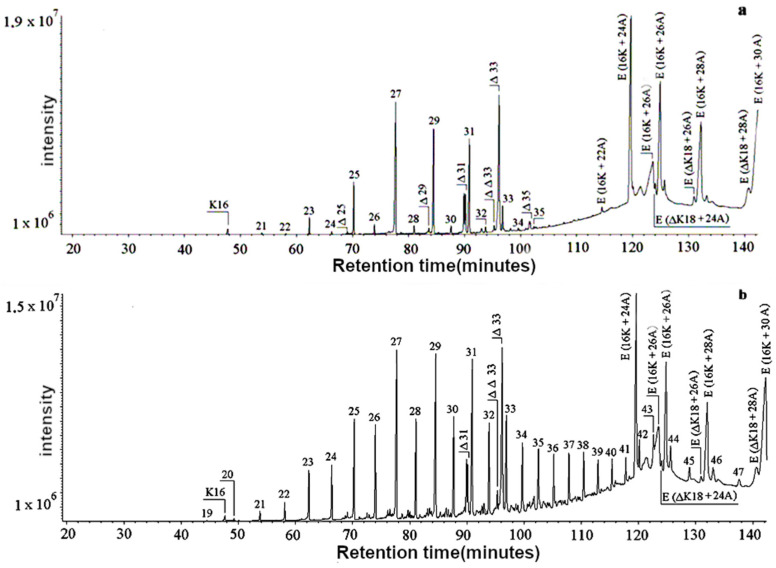
Total ion chromatogram of pure (**a**) and adulterated (**b**) beeswax samples analyzed by gas chromatography coupled with mass spectroscopy.

Gas chromatography coupled with mass spectrometry was used to identify differences between pure and adulterated beeswax samples. The obtained chromatograms are depicted in Figure 1.

In Figure 1, n-alkanes are designated according to the number of C-atoms; Δ represents n-alkenes with the appropriate number of C-atoms; ΔΔ represents n-alkadienes with the appropriate number of C-atoms; K represents saturated acids with the appropriate number of C-atoms; E (16K + 24A) represents esters of hexadecanoic acid and 1-tetracosanol; E (16K + 26A) represents esters of hexadecanoic acid and 1-hexacosanol, E (16K + 28A) represents esters of hexadecanoic acid and 1-octacosanol; E (16K + 30A) represents esters of hexadecanoic acid and 1-triacontanol; E (Δ18K + 24A) represents esters of oleic acid and 1-tetracosanol; E (Δ18K + 26A) represents esters of oleic acid and 1-hexacosanol; and (Δ18K + 28A) represents esters of oleic acid and 1-octacosanol. From Figure 1, it can be seen that pure beeswax contained alkanes with an odd C number, whereas the adulterated samples contained alkanes with an even C number and more than 35 carbon atoms. Also, the concentration of esters of palmitic acid and hexadocosonale decreased with the addition of the paraffin.

The exhibited chromatograms clearly show that with the addition of paraffin, the concentration of alkanes with even C numbers increased, whereas in the pure wax, only traces of these were observed.

### 2.3. Exploratory Analysis of Wax Samples Based on Physicochemical Characteristics

**Figure 2:**
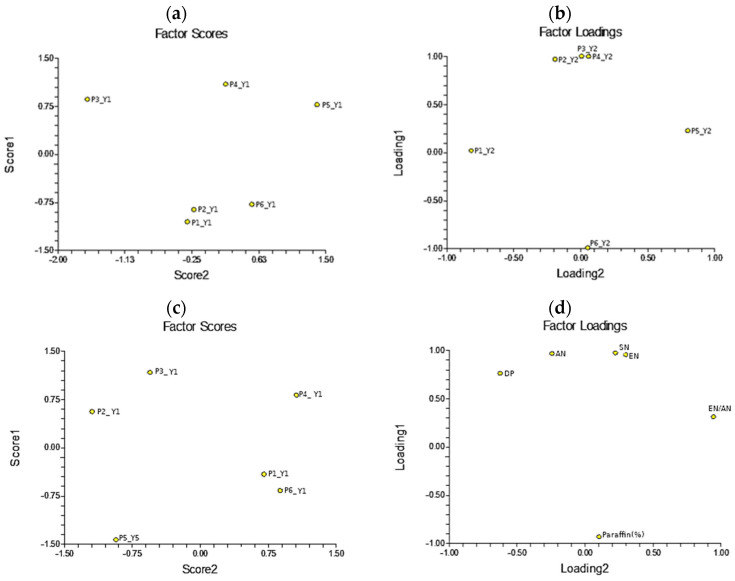
Principal component analysis for physicochemical parameters of wax samples collected during Y1 and Y2. Score graphs (**a**,**c**) and loadings (**b**,**d**) are shown for both years.

In order to examine the similarities and differences between different wax samples collected during Y1 and Y2, principal component analysis (PCA) was performed. In both cases, two-component models were obtained. The results depicted in Figure 2 show that for samples from Y1, principal components PC1 and PC2 described, respectively, 66.16% and 22.54% of the total variability in the data. In the case of the samples from Y2, PC1 and PC2 described 71.93% and 24.94% of the total variability in the data, respectively.

On the score graph (Figure 2a), it can be observed that the samples from Y1—P4, P5, and P3—had positive scores along the PC1 direction, while P1, P6, and P2 had negative scores. Observing the loading graph (Figure 2b), it can be concluded that the physicochemical parameters AN, SN, and EN would have dominant values in samples P4, P5, and P3, while the paraffin content would be elevated in the group comprising P1, P6, and P2. Also, for sample P3, a higher value of MP was expected compared to the rest, while for sample P4, a slightly higher value of EN/AN was expected compared to the others.

In the case of the Y2 wax patterns, the pattern grouping was somewhat different. Two groups with positive and negative scores along the PC1 component were still observed (Figure 2c). Similarly to the samples from Y1, producers P4 and P3 (positive scores) were expected to have elevated EN, SN, and AN values, while P6 and P1 were expected to have an elevated paraffin content (Figure 2d). However, in the case of producer P2 from Y2, a lower paraffin content and increased values of EN, SN, AN, and DP were expected (graph (d)). Also, the P5 producer from Y2 was expected to have a higher paraffin content and likely a higher DP, and lower values of EN, SN, and AN (graph (d)).

**Figure 3:**
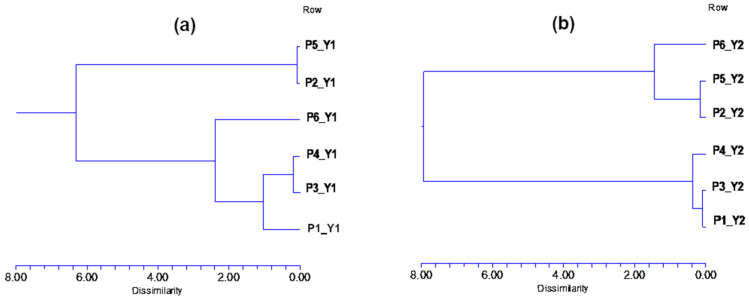
Hierarchical cluster analysis of wax samples collected during Y1 (**a**) and Y2 (**b**), based on physicochemical parameters.

Hierarchical cluster analysis (HCA) was used to investigate possible similarities in the properties of the foundation sheets. The diagrams are depicted in Figure 3.

HCA revealed two distinct clusters in year 1 and one subcluster in between. The first cluster pointed out similarity between P2 and P5, and the second was composed of the P4 and P3 subclusters with similar properties as P1. P6 showed specific properties as a separate cluster. On the other hand, in the second year, only two clusters were formed. The first one was composed of P1, P3, and P4, similarly to the cluster in year 1, with the onlyI confirm the changeI confirm difference being that P1 and P3 now formed a subcluster. The second one contained P2 and P5 as subclusters, but unlike in year 1, P6 belonged to this cluster also.

### 2.4. Multivariate Analysis of Variance—Influence of the Type of Producer on the Physicochemical Characteristics of Waxes

Multivariate analysis of variance (MANOVA) was used to examine the significance of the difference in physicochemical parameters of foundation sheets between different types of wax producers.

However, conducting testing separately for each year was not possible (zero degrees of freedom) due to the limited number of measurements taken (one measurement per parameter, per year, and per type of producer). Therefore, the combined results for the two-year period (Y1–Y2) were observed, and only direct linear members were included in the model, without interactions between factors.

The omnibus multivariate test showed that the influence of the type of producer was barely statistically significant (only according to Roy’s multivariate criterion), while there was no statistical significance for any of the differences between years. According to the established procedure, further univariate testing would not add any statistical value. Although certain differences could be observed in the average values of certain physicochemical parameters between the observed years, these differences were not statistically significant according to Tukey’s post hoc test (Honest Significant Difference (HCD)) where (*p* > 0.05), but they were according to Fisher’s post hoc test (Least Significant Difference (LCD)).

Regarding the differences in the average values of physicochemical parameters in relation to the type of producer based on the HCD test, no statistical significance can be observed (*p* > 0.05). But based on the LCD test for certain parameters, there were statistically significant differences in average values. Thus, in the case of the AN parameter, a difference can be observed between the average values for P6 in relation to P4 and P3 (LCD test, *p* < 0.05), while in the case of SN and EN, a statistically significant difference can be observed between the average values for P4 (in relation to P6 and P2, LCD test, *p* < 0.05) and P3 in relation to P2 (LCD test, *p* < 0.05). In terms of paraffin %, P6 and P2 differed significantly from P3 and P4, but not from the other samples (LCD test, *p* < 0.05).

### 2.5. Results of Testing the Acceptance of Foundation Sheets by the Bee Colony

Based on the physicochemical parameters, we investigated possible correlations between bees’ acceptance of foundation sheets and wax quality. The focus was on the presence of paraffin in the wax and how it affected the foundation sheets constructed in the hive. The research was carried out for two years in an experimental apiary; records were kept in detail for all parameters that were monitored. The data obtained for Y1 and Y2 are shown in Table 3 and Table 4.

Due to the low amount of nectar in Y1, worker bees produced a small quantity of wax. Hence, the monitoring of the construction of foundation sheets took a long time, despite the stimulation of the bee colonies with the sugar syrup they received daily.

After the linden began blossoming and the arrival of nectar in nature, the construction was accelerated, and immediately after the arrival of fresh nectar in nature, all colonies built foundation sheets and at the same time accepted the newly built wax, storing pollen and honey in them. It is very important to emphasize that the foundation sheets from the producers P2 and P6 did not serve as a basis for building wax cells or the rest of the frame, but the bees built irregular honeycombs on foundation sheets whose cells corresponded in diameter to the cells of the drone’s comb. In the case of foundation sheets from producer P2, on the fifth day of construction, the frame was accepted by the queen, and populated with a one-day-old brood. It was noted that the worker bees built cells for the drones on the aforementioned foundation sheets. The eggs were not fertilized. The brood consisted of drones, which hatched but were not directly useful for the expansion of the colony and its survival.

In Y2, under the same experimental conditions as in Y1, the construction took longer despite the constant stimulation of bee colonies with sugar syrup, both because of the limited nectar available in nature and the fact that there were several rainy days with a low air temperature during construction. The experiment lasted a total of 18 days, from the day the foundation sheets were placed in the hive to the bees’ acceptance of the constructed frames to store honey (H), pollen (P), and brood (B). Of the 18 days of the trial, 10 days were rainy. After the beginning of the linden blossoming and the arrival of nectar in nature, the construction was accelerated. All colony-built foundation sheets were accepted for the storing of pollen and honey.

### 2.6. Exploratory Analysis of Wax Samples Based on Acceptance Parameters

**Figure 4:**
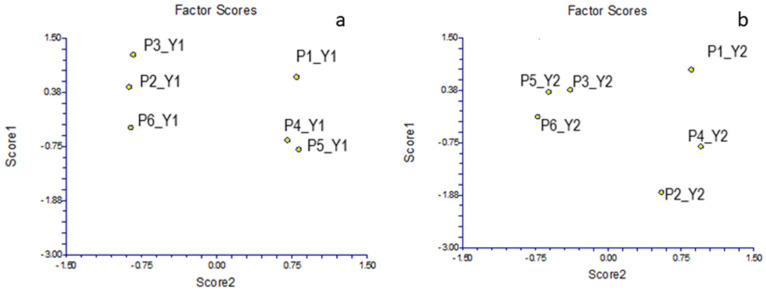
Principal component analysis (score graphs) based on acceptance parameters of wax samples collected during Y1 (**a**) and Y2 (**b**).

For the acceptance parameters, two-component models were obtained by principal component analysis. Score graphs for both years (Figure 4) indicate the existence of two groups of samples. The samples of producers P1, P4, and P5, observed during Y1, had positive scores, while P3, P2, and P6 had negative scores (Figure 4a), whereas P5 is positive in Figure 4b and negative in Figure 4a. Similarly, producers P1 and P4, which were observed during Y2, had positive scores, while P5, P3, P6, and P2 had negative scores, with P2 significantly standing out from the rest. Thus, during both years, the samples from producers P1, P4, P3, and P6 were grouped in a similar way and could be expected to show similar trends in acceptability during both years, while producers P5 and P2 showed significant differences in acceptability during Y1 and Y2.

**Figure 5:**
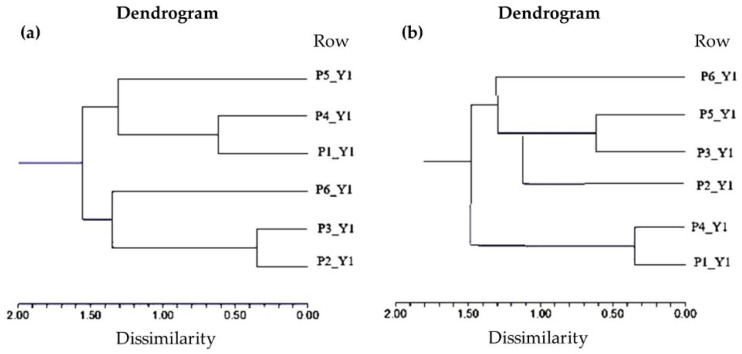
Hierarchical cluster analysis based on acceptance parameters of wax samples collected during Y1 (**a**) and Y2 (**b**).

Although in the case of PCA no graphs of loadings are provided, it should be noted that samples with positive scores have a higher degree of acceptability. Identical groupings can also be observed based on HCA, where in both cases two clusters can be observed at distances between 8 and 19 and 8.5 and 17.5 units (Figure 5).

## 3. Discussion

### 3.1. Assessments of Wax Quality Based on Physicochemical Parameters

Reference values for the melting point range from 61 °C to 66 °C. In all tested samples, the melting point was within the specified range, which may indicate that this parameter is the least sensitive to paraffin modification. As the paraffin content of beeswax increases, the melting point may rise or fall, depending on the melting point of the added paraffin. Namely, the softening point of the most common paraffin is 55–58 °C.

The acid number represents the content of free fatty acids in the beeswax, and the literature states that values from 17 to 24 correspond to natural waxes [1,20]. When the results obtained for the acid number are compared with the paraffin content, it can be seen that with the addition of paraffin, the value of the acid number decreased. In the samples we examined, the lowest value of the acid number was found for samples P6, P1, and P2 from Y1. Also, no sample corresponded to the reference interval, i.e., all had values of less than 17. It was found that there is a negative correlation between the paraffin content and the acid number, which is in agreement with other studies [16]. This trend is explained by the fact that the increase in the mass fraction of paraffin in beeswax decreased the amount of free fatty acids, because paraffin consists only of hydrocarbons, with the absence of free fatty acids.

The ester number represents the content of fatty acid esters and higher alcohols in the wax sample, and it is stated in the literature that values from 70 to 80 correspond to natural waxes. It was determined based on the difference between saponification and the acid number, and in all tested samples, the ester number was less than 70.

The saponification number represents the total content of free and fatty acid esters and higher alcohols in the wax sample, with values generally ranging between 87 and 104, which correspond to natural waxes [1,20]. In all tested samples, the saponification number was lower than 87.

The ratio of the ester and acid number represents the quotient of the value of the ester and acid numbers and ranges from 3.4 to 4.4 [2]. What is interesting is that the value of the ester number in all tested samples corresponded to literature data [1,20]. This indicates that this parameter is practically unsuitable for evaluating the modification of beeswax with paraffin.

The FTIR spectrum (position, intensity, number, and shape of absorption maxima) is directly related to the molecular structure and is very characteristic of each compound. Because the primary components of beeswax are monoesters, free fatty acids, and hydrocarbons, the most intense absorption bands in the infrared part of the spectrum come from these compounds. The band appearing at 1736 cm^−1^ originates from the valence vibrations of the C=O bond of the ester carboxyl group, while the band at wave number 1711 cm^−1^ originates from the valence vibrations of the C=O bond of the free carboxyl group. Paraffin characteristically does not absorb radiation in the area of 1750–1700 cm^−1^. In addition to these bands, the bands at 1470 cm^−1^ that come from CH_2_ deformations and at 1170 cm^−1^ from C–O vibrations are also characteristic. Using the appropriate calibration curve, it was possible to determine the percentage of paraffin in beeswax. In the tested samples, the paraffin content ranged from 19.75 to 85.68%. Bogdanov [1] states in his book on beeswax that the paraffin content in natural beeswax may reach a maximum value of 14%. However, he also notes that any presence of paraffin with an even number of carbon atoms indicates the modification of the wax with paraffin.

The risk of contaminated and/or adulterated beeswax to the health of honeybees was assessed by the Scientific Committee of the Federal Agency for the Safety of the Food Chain (FASFC) in advisory 18–2018 [34]. The Scientific Committee [20] has proposed limits of use for remelted beeswax placed on the market: the acid number of the wax should fall within 17 to 24, whereas the ester number of the wax should fall within 63 to 87.

Certain dependencies have been discovered between the physicochemical properties of beeswax and the degree of adulteration caused by the addition of paraffin [3,11,12,16,19]. As the amount of paraffin added to the beeswax increases, the density of the beeswax increases, and the acid and saponification numbers decrease. A higher addition of paraffin also results in a decrease in the value of the iodine index [23,29]. The boiling point of beeswax increases or decreases depending on the boiling point of the added paraffin [3,11,12,16,19].

Qualitative analysis was performed by gas chromatography coupled with a mass detector. The chromatograms obtained for pure beeswax and paraffin exhibit distinctive differences, especially in the increasing peak intensity for even numbered alkanes [9,11,21,22,23,24,25,26,27], and such analyses turned out to be very useful in detecting adulteration. The intensity of these peaks increased when the addition of paraffin to the beeswax was increased.

The adulteration of beeswax with fatty products can be detected either by modifying the concentrations of beeswax compounds or by detecting, in chromatograms, compounds that are not present in beeswax [27]. Beef tallow is mainly composed of a series of triacylglycerols with high molecular weights. These tri-ester compounds are not easily observed by GC, but a chromatogram of a tallow solution in chloroform showed the presence of free acids, which were not detected in the beeswax chromatograms.

In 2003, Jiménez et al. [25], using GC and mass spectrometry detection, determined the fractions of alcohol and acids present in pure beeswax from *Apis mellifera*. During their research, they concluded that commercial beeswax foundations had different chromatographic profiles than natural beeswaxes, probably as a result of mixing them with other, cheaper substances during the production process. These foreign substances are responsible for modifying the content of naturally occurring fatty compounds, increasing their concentrations, and even causing the loss of smaller compounds if excessive dilution occurs. A chromatogram of typical pure beeswax, after specific sample preparation, can be used as an imprint to ensure the quality of commercial beeswax. Observing extraneous chromatographic peaks or changes in the relative amounts of beeswax compounds can be used to distinguish between adulterated beeswax foundation sheets [25].

The paraffin content in the samples of beeswax foundation sheets determined by FTIR–ATR spectroscopy differs from the content estimated on the basis of the values of physicochemical parameters. Such deviations may be the result of physicochemical changes that take place during wax recycling at high temperatures. Heating the wax at a temperature higher than 100 °C results in a decrease in the acid number, an increase in the ester number, and a decrease in the content of unsaturated hydrocarbons [23]. It should be taken into account that the addition of certain substances (paraffin and microcrystalline wax) contributes to a reduction in the concentration of free acids and esters in the wax, while the addition of some other substances (fats of animal origin, stearic acid and its esters) results in their increase. The samples, whose physicochemical parameters were found within the framework prescribed by the legal regulations for pure beeswax, should be examined using instrumental techniques that provide the possibility of detecting all their components. The paraffin content in foundation sheets determined on the basis of the ester/acid ratio was significantly different from the paraffin content determined using other methods. This parameter is extremely sensitive to experimental conditions because it is calculated based on two experimentally determined values, so it cannot be a reliable indicator of beeswax quality. The results led to the conclusion that the determination of the softening point is not a suitable method for estimating the mass fraction of paraffin in wax. Due to the addition of paraffin and synthetic materials whose softening point values are higher than those characteristic of pure beeswax, the constructed model did not ensure the determination of paraffin content in a large number of tested samples [30].

Bernal et al. [16] reported that the acid value of a mixture decreased linearly with an increase in the percentage of adulterants. A significant increase in the acid value was observed when the adulteration percentage increased, in a linear manner.

The saponification number decreased when the beeswax was mixed with paraffin, while the same value increased in adulterations with tallow. Tallow and stearic acid were the products that most affected the change in the ester number. As expected, the only parameter that changed was the melting point, depending on what the beeswax was adulterated with. The melting point decreased when beeswax was mixed with increasing amounts of tallow and paraffin, with lower melting points that were in the ranges of 54–56 °C and 58–60 °C [16].

Based on the quality control results for beeswax presented in reports by other authors [12,17], it must be noted that there is a constant problem with the adulteration of beeswax. This may be due to the lack of mandatory regulations related to the quality of beeswax and the insufficient scale of beeswax production [23].

In 2007, the European Food Safety Authority (EFSA) adopted a scientific opinion on the use of beeswax as a food additive (E901), as a glazing agent, and as a flavor carrier [4]. Paraffin, the material most often used to adulterate beeswax, is approved for use as a material in plastics for food contact material (FCM 93), according to Commission Regulation 10/2011 [35] for the category waxes, paraffinic, refined, derived from petroleum or synthetic hydrocarbon raw materials, or of low viscosity [36].

There are currently no guidelines available for beekeepers that provide ways of recycling beeswax. Several good beekeeping manuals suggest guidelines for good beekeeping practice [37,38,39,40].

### 3.2. Acceptance of Foundation Sheets by the Bee Colony

While planning and setting up the experiment, which served to evaluate the acceptance of the wax foundation sheets by the bee colony, it was ensured that the bee colonies under study were of equal strength, with the same amount of brood, food, and bee workers. The conditions were constant for all six bee colonies. During the experiment, bee colonies were stimulated daily with sugar syrup (1:1 sugar to water ratio). All six bee colonies were in the same location, so the weather conditions were the same, as was the composition of the natural flower pasture. The experiment was carried out over two years in the same period. The most suitable time for experimenting was after the end of extracting honey from acacia, after acacia pasture, at the end of May and the beginning of June. This time was chosen because at that point, the bee colonies slowly come out of their reproductive cycle, dedicating their energy to collecting the small amount of nectar that is present at that time, and the beekeepers are slowly preparing them for the beginning of the linden blossoming.

The duration of the research carried out in Y1 was a total of 20 days from the day of the frames with foundation sheets were placed in the bee colony to the moment of their complete construction, which subsequently results in the utilization of these new cells for storing honey, pollen, and brood. The experiment was set up in the same way during Y2 and lasted 18 days from the day of the frames with foundation sheets were placed in the bee colony to their complete construction and start of use. It is very important to emphasize that during the research in Y1, all 20 days were sunny with a small (insignificant) amount of nectar intake from nature. During the research in Y2, out of the 18 days of the research, 10 days were rainy days with bad weather conditions, preventing the bees from leaving the hive to collect food and water.

By observing the quality of wax cells and the rate of construction in the research from Y1, the sample foundation sheets P1, P4, and P5 proved to be the samples that were first accepted by the bee colony. From the moment they were placed in the hive, it took the bees six days to build the foundation sheets and store honey and pollen in them. Sample P3 was very well received at the start, and the construction proceeded as with the previously mentioned samples. It took 9 days for the bees to reach 80% of the built-up wax spread on the frame and 50% of the built-up depth of the wax cells. Eventually, construction slowed down, and it took the colony another 11 days to complete the spread (20%) and depth of wax cells (50%). Samples P2 and P6 were poorly received from the very beginning, and construction went much worse than with the other samples. After the second day in the case of sample P6 and the eleventh day in the case of sample P2, the construction of an irregular honeycomb was observed. The cells were deformed and crooked, with several cells joined into one, and the bees did not follow the printed matrix on the foundation sheet, and the size of the cells did not correspond to the size of the worker honeycomb (the cells were significantly larger than the worker cells—the so-called drone cells). The disadvantage of such deformed cells is reflected in the fact that the strength of the constructed frame was significantly impaired, and the frames broke.

Developing brood from combs with such cells does not have a positive effect on the development of the bee colony, because the queen lays only unfertilized eggs in drone cells, from which drones develop. For samples P2 and P6, the construction process lasted 20 days. Regardless of the percentage of paraffin, all samples were accepted. In the case of two samples with a high percentage of paraffin (P2 and P6), a difference was observed in the rate of construction and the quality of the upgraded wax cells compared to the samples with a low percentage of paraffin. In the case of sample P1, this cannot be said, considering that the sample was well accepted by the bee colony and contained a high percentage of paraffin. The time when these foundation sheets were built coincides with the beginning of the linden blossoming and the arrival of a large amount of nectar in nature.

Based on the quality of the wax cells and the rate of construction in the research from Y2, all six samples were initially well received by the bee colony. The frames were built equally with small deviations in the P6 sample. There were no deformed cells. The rate and quality of construction were better compared to those built in Y1. The aggravating circumstance in the Y2 research was the weather. The varying episodes of rain and sun from day to day made it much more difficult for the bees to leave the hive. During the period from the 11th to the 16th day of the experiment, it rained every day. During these bad weather conditions, the linden blossomed, but there was no nectar in nature. After the rain stopped on the 17th day, the intake of nectar from nature was recorded as 2.5 kg per hive. On the very next day, the foundation sheets were completely built, and honey was already stored in them.

During their study, Jiménez et al. [26] concluded that the monitored parameters in the examined foundation sheets of beeswax accepted by the bees were within the range of appropriate orientation values in the majority of samples. Only small deviations were found in the concentrations of several minor compounds. The rejection of beeswax foundation sheets by bees cannot be exclusively related to an increase or decrease in the concentration of chemical compounds found in the wax [11].

## 4. Materials and Methods

### 4.1. Sample Collection

From the six manufacturers that are most represented on the market in Vojvodina Province, Serbia, 2 kg of beeswax foundation was purchased and used in the research within the framework of this paper. Depending on the average weight of one foundation sheet, 2 kg contains 22 to 29 foundation sheets. Six foundation sheets from each manufacturer were used to assess the quality of the wax. Two foundation sheets from each manufacturer were used to evaluate the acceptance of the foundation sheets by the bees. The remaining foundation sheets were saved for the control sample.

### 4.2. Experiment in Apiary

The beeswax foundation sheets, whose quality was examined in this research, were subjected to an examination to assess their acceptance by the bee colonies. The experiment was conducted at the experimental apiary in Susek, in the municipality of Beočin, on the northern slopes of Fruška Gora, geo. coordinates 45.216625/19.528879. The optimal time for setting up the experiment was in the period between acacia and linden pasture.

The test was carried out in two consecutive years, the first (Y1) and second year (Y2), at the same time interval.

At the very beginning of the research, the number of bee colonies necessary for the research was defined. For every foundation sheet sample producer, there was one bee colony, with a total of six bee colonies. These six healthy bee colonies had the same amount of brood and food, and the same number of bees. Two foundation sheets from each tested producer were previously poured into wired frames of the Langstroth–Ruth (LR) type, and then the frames were placed in the already-prepared bee colonies. The position of the frames, in the hive body between the last frame of the brood and the frame on which the pollen was located, and on both sides of the brood, was the same for all six bee colonies. After placing the foundation sheets in the bee colonies, every 24 h, the construction of the foundation sheets was checked and assessed based on the number of built cells and their appearance. A fully built LR frame contains 9600 cells on both sides. The point at which the bees began to store nectar or when the queen began to lay eggs in the built-up wax was also monitored. All changes to the frames were properly photographed, recorded, and noted down in tables.

The control experiment was carried out until the observed foundation sheets were built to more than 90% completion. Throughout the duration of the experiment, sugar syrup was given to the bee colonies at the amount of 500 mL per day per bee colony. Sugar syrup was given to bee colonies because with this additional amount of food, the rate of construction would be increased, and, in addition, this additional food would compensate for the lack of nectar in nature due to bad climatic conditions. The experiment lasted 18 days.

### 4.3. Sample Preparation

The collected foundation sheets were analyzed directly. Briefly, 50 mg of representative beeswax sample was dissolved in 5 mL of chloroform and treated with ultrasound for 10 min at 50 °C. An aliquot of 1.5 mL was transferred to vials and kept for GC–MS analysis.

### 4.4. Reagents

All reagents used to determine physicochemical parameters, GC–MS, and FTIR analysis were of analytical grade. Potassium hydroxide hydrochloric acid, ethanol, paraffin, and phenolphthalein were obtained from Merck, Darmstadt Germany.

### 4.5. Determination of Physicochemical Parameters

Drop point, acid, ester, and saponification assays were used to examine the quality of beeswax. All measurements were performed in duplicates.

#### 4.5.1. The Drop Point

The drop point was determined by the ring method, for which the beeswax was melted and put into a ring. After solidification, the excess was removed with a spatula and left at ambient temperature for at least 18 h. The ring was then transferred to a water bath and slowly heated. The drop point represents the temperature at which a ball passes through the ring and is expressed in °C.

#### 4.5.2. The Acid Number

The acid number (value) is defined as the amount of (KOH) in milligrams required to neutralize a gram of the wax. The acid number (value) was determined by the procedure published in *European Pharmacopoeia*, 10.0, with a slight modification [20]. Briefly, 3.00 g of sample was transferred to a 250 mL conical flask fitted with a reflux condenser containing 40 mL of xylene and a few glass beads. The flask was heated until the sample dissolved. Then, 20 mL of ethanol and 0.5 mL of 1% phenolphthalein solution were added, and the hot solution was titrated with 0.5 mol L^−1^ alcoholic potassium hydroxide until a red color persisted. The acid number was calculated using the expression:

AN = [56.1(V − V_0_)c]/m

where V—volume of KOH (mL) used for titration of analyte; V_0_—volume of KOH (mL) used for titration of blank; c—concentration of KOH (mol L^−1^); m—mass of analyte (g).

#### 4.5.3. The Saponification Number

The saponification number (value) is defined as the amount of KOH in milligrams needed to neutralize 1 g of beeswax after saponification, i.e., alkaline hydrolysis. Briefly, 2.00 g of wax sample was weighted and transferred to a 250 mL conical flask fitted with a reflux condenser. Then, 30 mL of alcohol and 30 mL of xylene were added. The flask was heated in a water bath until the sample was dissolved. Then, we added 25.0 mL of 0.5 mol L^−1^ alcoholic potassium hydroxide and refluxed it for at least 4 h. The hot solution was immediately titrated with 0.5 mol L^−1^ hydrochloric acid, using 1 mL of 1% phenolphthalein as an indicator. The endpoint for the titration was reached when the red color disappeared. A blank was set up by following the same procedure but in this case without a sample. The saponification number was calculated using the expression:

SN = [56.1(V_0_ − V)c]/m

where V—volume of KOH (mL) used for titration of analyte; V_0_—volume of KOH (mL) used for titration of blank; c—concentration of KOH (mol L^−1^); m—mass of the analyte (g).

#### 4.5.4. The Ester Number

The ester number (value) is defined as the amount of KOH in milligrams needed to neutralize 1 g of ester-linked acids of beeswax. The value was calculated from differences between the saponification and acid numbers.

### 4.6. GC–MS Method

Sample preparation: 50 mg of representative beeswax sample was dissolved in 1.5 mL of chloroform and ultrasonicated for 10 min.

A Clarus 680 Perkin Elmer chromatograph equipped with a Clarus SQ8T mass spectrometer (Perkin Elmer PerkinElmer, Inc., Waltham, MA, USA) was used for GC–MS analysis. The gas chromatographic separation was achieved using a nonpolar column HP-5MS, 30 m × 0.25 mm, film thickness 0.25 μm. Helium was used as the carrier gas, and its flow rate was set at 1.5 mL/min. Beeswax samples were dissolved in chloroform (1 mg/30 μL), and the volume of the sample solution injected into the column was 1 μL.

The separation of components was carried out according to the following temperature regime: increase from 80 °C to 320 °C with an increment of 2 °C/min; 320 °C maintained for 20 min; increase from 320 °C to 323 °C with an increment of 2 °C/min; 323 °C maintained for 1 min (total time 142.5 min). The temperature of the ion source was 230 °C, and the quadrupole was 150 °C. Analysis was performed in scan mode, with scanning undertaken in the range from 50 to 650 U.

Individual peaks were identified based on the interpretation of mass spectra and their comparison with the NIST5a library. In addition to TIC (Total Ion Current), for identification, typical ion fragmentograms were used: *m*/*z* 71 for n-alkanes; *m*/*z* = 73 for fatty acids; *m*/*z* = 257 for hexadecanoic acid esters; *m*/*z* = 264 for oleic acid esters; and *m*/*z* = 88 to determine the presence of ethyl esters of fatty acids.

### 4.7. FTIR Spectroscopy

The FTIR–ATR technique was used for beeswax authentication and the detection of adulteration in collected beeswax samples.

The calibration standard was prepared from pure beeswax to pure paraffin with an increment of 5%. The paraffin content was determined from the correlation of calibration standards and peak intensities at 1736, 1470, and 1170 cm^−1^.

The Thermo Scientific Nicolet 6700 FTIR spectrometer, using the ATR technique from the smart accessory and diamond crystal (Smart Orbit, Thermo Scientific, Madison, WI, USA), was used for IR spectral measurements. Spectral data were collected in the mid-IR range (4000–400 cm^−1^) with 32 scans and a 4 cm^−1^ resolution. A background spectrum was recorded with 32 scans. IR spectra were baseline-corrected using OMNIC software (version 7.0, Thermo Scientific, USA) and exported to SPC files for statistical analysis.

### 4.8. The Statistical Analysis

The results were normalized and standardized before analysis. Principal component analysis (PCA) was performed using PLS ToolBox (http://www.eigenvector.com/software/pls_toolbox.html accessed on 14 November 2024). PCA was carried out on standardized data using a singular-value decomposition NILPS algorithm, and a 0.95 confidence level was considered as statistically significant. Hierarchical cluster analysis was performed on normalized data using the Euclidian distance as a measurement of distance. The clusters were organized by the Ward method. MANOVA was performed using the simplest linear correlation on physicochemical parameters. In both instances, a sequential model of total variance was used.

## 5. Conclusions

The presence of paraffin in the range of 19.75 to 85.68% was detected in the examined samples of wax foundation sheets.

The presence of beef tallow was not detected during the two years of research. Adulteration with paraffin was expected, considering that most researchers who dealt with this topic also detected paraffin as a substance used to adulterate beeswax.

Monitoring the acceptance of the newly introduced foundation sheets during the research proved that without quality nectar in nature, there is no intensive building of wax. During Y1, high percentages of paraffin were detected in samples from three manufacturers (P1, P2 and P6), and samples from manufacturers P2 and P6 had the worst/slowest acceptability, and the quality of the built frames was very poor. A large percentage of drone cells were built and cells were irregularly placed in relation to the given template of the printed base. During Y2, the speed of construction for the inserted foundation sheets was the same for all samples, and the percentages of present paraffin were approximate. During the research in Y2, the atmospheric conditions were much worse, and the influence of bad weather on the speed of the construction of wax frames was visible.

## Figures and Tables

**Table 1:** The physicochemical profile of the analyzed wax foundation sheets.

Foundation Sheet ^1^	Year Parameters ^2^	DP (°C)	AN	SN	EN	EN/AN	Paraffin (%)	Beef Tallow (%)
P1	Y1	62.3 ± 3.5	3.8 ± 0.3	17.5 ± 2.1	13.8 ± 1.3	3.66 ± 0.37	82.7 ± 9.8	ND ^3^
	Y2	61.7 ± 3.5	12.9 ± 0.7	62.1 ± 7.4	49.2 ± 4.8	3.82 ± 0.39	37.0 ± 4.4	ND
P2	Y1	62.0 ± 3.5	5.6 ± 0.3	25.0 ± 3.0	19.4 ± 1.9	3.49 ± 0.36	85.7 ± 10.2	ND
	Y2	64.3 ± 3.6	15.3 ± 0.9	67.6 ± 8.0	52.3 ± 5.1	3.41 ± 0.35	32.4 ± 3.9	ND
P3	Y1	63.2 ± 3.5	14.5 ± 0.8	65.1 ± 7.7	50.6 ± 4.9	3.50 ± 0.36	22.2 ± 2.6	ND
	Y2	63.8 ± 3.6	15.8 ± 0.9	71.6 ± 8.5	56.8 ± 5.5	3.60 ± 0.37	21.6 ± 2.6	ND
P4	Y1	61.4 ± 3.4	15.9 ± 0.9	72.8 ± 8.7	56.9 ± 5.5	3.58 ± 0.37	19.8 ± 2.4	ND
	Y2	62.9 ± 3.5	14.4 ± 0.8	73.0 ± 8.7	58.6 ± 5.7	4.06 ± 0.41	29.9 ± 3.6	ND
P5	Y1	61.8 ± 3.5	10.7 ± 0.6	63.8 ± 7.6	53.1 ± 5.2	4.96 ± 0.51	30.0 ± 3.6	ND
	Y2	62.1 ± 3.5	11.6 ± 0.6	49.8 ± 5.9	38.3 ± 3.7	3.31 ± 0.34	40.9 ± 4.9	ND
P6	Y1	61.8 ± 3.5	5.0 ± 0.3	24.9 ± 3.0	19.9 ± 1.9	4.02 ± 0.41	79.2 ± 9.4	ND
	Y2	61.2 ± 3.4	12.5 ± 0.7	60.3 ± 7.2	47.8 ± 4.6	3.83 ± 0.39	38.6 ± 4.6	ND
References	[20]	61–66	17–22	87–102	70–80	3.3–4.3 [2]	without presence	without presence

Abbreviation: ^1^ P1–P6—different producers, ^2^ Y1—first year, Y2—second year; ^3^ ND—not detected.

**Table 2:** The detected paraffin content values in samples determined by FTIR–ATR analysis.

Foundation Sheet		Abs on 1736	Abs on 1470	Abs on 1170	Paraffin (%)
P1	Y1	0.0315	0.1261	0.0269	82.7 ± 9.8
	Y2	0.1036	0.1105	0.0969	37.0 ± 4.4
P2	Y1	0.0269	0.1135	0.0177	85.7 ± 10.2
	Y2	0.1108	0.1337	0.0567	32.4 ± 3.9
P3	Y1	0.1269	0.118	0.0862	22.2 ± 2.6
	Y2	0.1279	0.1123	0.0782	21.6 ± 2.6
P4	Y1	0.1307	0.1191	0.0969	19.8 ± 2.4
	Y2	0.1148	0.1426	0.0455	29.9 ± 3.6
P5	Y1	0.1146	0.1249	0.0904	30.0 ± 3.6
	Y2	0.0974	0.1308	0.0506	40.9 ± 4.9
P6	Y1	0.0372	0.1427	0.0391	79.2 ± 9.4
	Y2	0.1011	0.1024	0.0410	38.6 ± 4.6

**Table 3:** Assessment of the acceptance of foundation sheets by the bee colony during Y1.

Foundation Sheet	Monitored Parameters of the Frame	Day 1		Day 2		Day 3		Day 4	
		Left Frame	Right Frame	Left Frame	Right Frame	Left Frame	Right Frame	Left Frame	Right Frame
P1	E (%)	95	90	95	90	95	90	100	95
	C (%)	30	20	40	30	50	60	90	90
P2	E (%)	5	10	5	10	20	20	60	30
	C (%)	0	5	0	5	10	10	40	30
P3	E (%)	35	15	35	20	50	30	65	40
	C (%)	20	10	25	10	30	20	50	30
P4	E (%)	0	40	5	40	30	70	90	90
	C (%)	0	25	10	25	25	50	70	70
P5	E (%)	0	10	10	25	40	60	80	95
	C (%)	0	5	5	50	50	50	90	90
P6	E (%)	0	20	0	30	0	30	0	40
	C (%)	0	10	0	10 ^1^	0	10 ^1^	0	30 ^1^
		Day 5		Day 6		Day 7		Day 8	
P1	E (%)	100	95	100	95	100	100	100	100
	C (%)	90	90	100 ^2^	100 ^3^	100 ^2^	100 ^3^	100 ^2^	100 ^3^
P2	E (%)	60	50	70	50	70 ^1^	50	70 ^1^	80
	C (%)	40	50 ^4^	60	80 ^4^	70	80 ^4^	80	90 ^4^
P3	E (%)	65	40	80	65	80	65	80	75
	C (%)	50	30	50	50	50	50	75	70
P4	E (%)	95	95	95	95	95	95	95	95
	C (%)	90	90	100	100	100	100	100	100
P5	E (%)	90	95	100	100	100	100	100	100
	C (%)	95	90	95	95	95	95	95	95
P6	E (%)	40	75	60	85	60	85	70	85
	C (%)	35	55 ^1^	45	80 ^1^	45	80 ^1^	55	80 ^1^
		Day 9		Day 10		Day 11			
P1	E (%)	100	100	100	100	100	100	100	100
	C (%)	100 ^2^	100 ^3^	100 ^2^	100 ^3^	100 ^2^	100 ^3^	100 ^2^	100 ^3^
P2	E (%)	90 ^1^	80	90 ^1^	90	90^1^	90	90 ^1^	80
	C (%)	90	90 ^4^	90	90 ^4^	90	90 ^4^	90	90 ^4^
P3	E (%)	80	75	85	85	85	85	80	75
	C (%)	75	70	85	85	85	85	75	70
P4	E (%)	95	95	95	95	95	95	95	95
	C (%)	100	100	100	100	100	100	100	100
P5	E (%)	100	100	100	100	100	100	100	100
	C (%)	95	95	95	95	95	95	95	95
P6	E (%)	100	100	100	100	100	100	100	100
	C (%)	90	90 ^1^	90	90	90	90	90	90 ^1^

^1^ Upgraded irregular honeycomb; ^2^ presence of pollen in wax cells; ^3^ presence of honey in wax cells; ^4^ presence of brood in wax cells; E—expansion; C—cell depths.

**Table 4:** Assessment of the acceptance of foundation sheets by the bee colony during Y2.

Foundation Sheet	Monitored Parameters of the Frame	Day 1		Day 2		Day 3		Day 4	
		Left Frame	Right Frame	Left Frame	Right Frame	Left Frame	Right Frame	Left Frame	Right Frame
P1	E (%)	45	17	It rained for the prior 24 h. The appearance of the clock bases did not change compared to the previous review.		45	17	It rained for the prior 48 h. The appearance of the clock bases did not change compared to the previous review.	
	C (%)	50	20			80	50		
P2	E (%)	90	85			90	85		
	C (%)	50	50			70	70		
P3	E (%)	5	5			5	5		
	C (%)	30	30			30	30		
P4	E (%)	80	60			80	80		
	C (%)	70	60			80	80		
P5	E (%)	10	10			25	10		
	C (%)	20	15			40	30		
P6	E (%)	10	10			10	10		
	C (%)	20	20			25	25		
		**Day 5**		**Day 6–10**		**Day 11**			
P1	E (%)	75	40	For the period from 06.19 to 06.24, it was constantly raining (the linden blossomed, but nectar in nature was absent due to the bad weather). The first sunny day was 25. 06, when an intake of 2.5 kg of natural nectar was recorded.		95 ^3^			
	C (%)	80	55						
P2	E (%)	95	95			95 ^3,4^			
	C (%)	70	95						
P3	E (%)	40	30			85 ^3^			
	C (%)	60	50						
P4	E (%)	95	80			80 ^3^			
	C (%)	95	90						
P5	E (%)	50	25			95 ^3^			
	C (%)	50	30						
P6	E (%)	25	20			60 ^3^			
	C (%)	50	50						

^3^ presence of honey in wax cells; ^4^ presence of brood in wax cells; E—expansion; C—cell depths.

## Data Availability

Data are contained within the article.
